# Disparities in Stress Exposure and Later-Life Disability

**DOI:** 10.1093/geroni/igae039

**Published:** 2024-04-25

**Authors:** Madison R Sauerteig-Rolston, Kenneth F Ferraro

**Affiliations:** Department of Sociology, Purdue University, West Lafayette, Indiana, USA; Center on Aging and the Life Course, Purdue University, West Lafayette, Indiana, USA; Department of Sociology, Purdue University, West Lafayette, Indiana, USA; Center on Aging and the Life Course, Purdue University, West Lafayette, Indiana, USA

**Keywords:** Activities of daily living, Cumulative stress burden, Ethnicity, Nativity, Race

## Abstract

**Background and Objectives:**

Drawing from cumulative inequality theory and the weathering hypothesis, this study examined the relationship between life-course stress exposure (measured cumulatively and by domains) and the onset of disability in later life among White, Black, U.S.-born Hispanic, and foreign-born Hispanic older adults.

**Research Design and Methods:**

Cross-sectional and longitudinal models were estimated using nationally representative data from the Health and Retirement Study (*N* = 11,483). We used logistic regression models to examine associations between stress exposure and Wave 1 disability (i.e., occurrence), and Weibull-accelerated failure-time models to examine the relationship between stress exposure and age of onset of disability 12–14 years later (i.e., incidence). We tested for moderation between stress and disability by race, ethnicity, and Hispanic nativity.

**Results:**

At Wave 1, higher odds of disability occurrence were associated with cumulative stress burden (CSB; odds ratio [OR] = 4.93, 95% confidence interval [CI]: 2.95–8.23). In a model specifying domains of stressors, disability occurrence was associated with childhood financial strain (OR = 1.22, CI: 1.01–1.46), lifetime traumatic events (OR = 1.92, CI: 1.41–2.62), neighborhood disadvantage (OR = 1.32, CI: 1.01–1.73), and major lifetime discrimination (OR = 1.64, CI: 1.12–2.41). Over time, earlier onset of disability was associated with CSB (β = −0.39), childhood traumatic events (β = −0.16), adult financial strain (β = −0.17), everyday discrimination (β = −0.15), and major lifetime discrimination (β = −0.13). The effect of childhood traumatic events on the transition to disability was stronger for U.S.-born Hispanic adults than White adults (occurring 33% earlier).

**Discussion and Implications:**

To reduce racial, ethnic, and nativity disparities in disability, it is important to consider the historical and structural disadvantages associated with stress exposure across the life course. It is also important to acknowledge that nativity influences stratification processes associated with disparities in racial and ethnic health trajectories.

**Translational Significance:** Exposure to stress across the life course is associated with an earlier onset of disability in later life, and the effect is especially detrimental for U.S.-born Hispanic adults. To reduce disparities in the onset and burden of disability among older adults, interventions should address lifetime stress accumulation, including the long-term consequences of childhood traumatic events and discrimination.

Disability, defined as restrictions in an individual’s ability to perform basic self-care tasks, is a threat to all older adults (Verbrugge & Jette, 1994), but evidence reveals that Black and Hispanic adults are at notably higher risk of experiencing disability than White adults (Fuller-Thomson et al., 2009; Garcia et al., 2017; Hayward et al., 2014; Vásquez et al., 2020). One plausible explanation for the disparities in disability is the impact of accumulated stressors (Ferraro et al., 2017; Geronimus et al., 2006; Williams et al., 2019). Stressors over the life course may accelerate health decline, but there is a need to identify stressors associated with disability onset and whether their influence is moderated by race, ethnicity, and nativity.

Guided by cumulative inequality theory (CIT) and the weathering hypothesis, this study uses longitudinal data from a diverse national sample to examine how cumulative stress burden (CSB) across the life course, as well as an inventory of domain-specific stressors, are associated with later-life disability among older adults. This study examines the prevalence of stress exposure and disability among four racial, ethnic, and Hispanic nativity groups: U.S.-born non-Hispanic White (hereafter, White), U.S.-born non-Hispanic Black (hereafter, Black), U.S.-born Hispanic, and foreign-born Hispanic older adults. Using both cross-sectional and longitudinal analyses, this study investigates how stress exposure is associated with the occurrence and incidence of disability across a 12- to 14-year study period. Finally, this study tests interactions between stress exposure and race, ethnicity, and Hispanic nativity (hereafter, nativity) to identify stressors associated with disability among different groups.

## Disparities, Stress, and Disability

A large body of literature reveals that Black and Hispanic people are more likely than White people to be exposed to varied types of stressors including early-life stressors (Slopen et al., 2016; Vásquez et al., 2019); lifetime traumas (Sauerteig et al., 2022); perceived discrimination (Vásquez et al., 2019); financial strain (Brown et al., 2020); and disadvantaged neighborhoods (Williams & Collins, 2001). Although these studies have greatly enhanced our understanding of stress processes by race and ethnicity, there are two notable gaps in the literature on whether stressors raise the risk of disability. First, Boen (2020) notes that most studies of stress exposure and health tend to examine the association between a single domain of stress and a health outcome. There is a critical need to better understand how the *cumulative* burden of multiple insults operates across the life course (Ferraro & Morton, 2018). To our knowledge, however, no study examines the cumulative burden of a large inventory of potentially stressful experiences as well as distinct domains of stress to understand how these experiences may proliferate and exacerbate racial and ethnic inequalities in later-life disability.

Second, relatively few studies examine how race, ethnicity, and nativity may moderate the relationship between stressors and disability. Given their distinct life experiences, we ask whether the association between stress and disability differs based on race, ethnicity, and nativity. Although minority populations are exposed to higher levels of cumulative stress (Boen, 2020; Chen et al., 2022; Sternthal et al., 2011), this does not necessarily lead to worse health outcomes. Brown and colleagues (2020) found that despite experiencing more stressors, Black and Hispanic Americans were less likely to be upset by these exposures. Closely related to disparities by race and ethnicity is the dearth of systematic research on the role that nativity plays in the development of health outcomes. Scholars argue that it is imperative to account for nativity among Hispanic Americans when examining health outcomes because not doing so “risks masking substantial heterogeneity within and across groups” (Boen & Hummer, 2019; p. 435; Hayward et al., 2014; Sternthal et al., 2011; Torres, 2015). Given the high stress levels and socioeconomic disadvantage reported by many Hispanic adults, it has been considered paradoxical that their health status is comparable to that of non-Hispanic White people (Markides & Coreil, 1986). Scholars caution against essentializing tendencies for race and ethnic groups because intersecting domains of identity—including nativity—create unique *lived experiences* (Torres, 2015). Failure to consider nativity may lead to an inaccurate picture of stress exposure and its influence on health among Hispanic people.

Based on the current literature on the relationship between demographic groups, stress exposure, and disability, we aim to (a) identify racial, ethnic, and nativity disparities in stress exposure, both cumulatively and domain-specific (e.g., childhood traumatic events, major lifetime discrimination) and rates of disability, (b) identify which type of stressors may be most harmful for disability onset among older adults, and (c) examine how the relationship between stress exposure and disability varies by race, ethnicity, and nativity.

## Theoretical Framework and Hypotheses

The present study is guided by CIT and the weathering hypothesis. CIT is a middle-range theory that integrates ideas from the life-course perspective, stress accumulation, and cumulative advantage/disadvantage to highlight the ways in which stress processes shape later-life health trajectories through exposure to accumulated risks and resources (Ferraro & Shippee, 2009; Ferraro et al., 2009). The theory specifies that social systems generate inequality, which is manifested over the life course through developmental and demographic processes (Ferraro et al., 2009). Inequality is systematically structured and embedded in society, with some people having disproportionately higher exposure to disadvantaged life circumstances. Racism is a system that generates inequality by (a) categorizing people into ranked social groups and (b) allocating differential access to risks, resources, and opportunities to groups defined as lower ranked. It is a form of systemic inequality that has plagued society for hundreds of years. Although overt forms of racism such as Jim Crow Laws are illegal, it is imprudent to assume that racism is a thing of the past. In fact, Desmond and Emirbayer (2009) described contemporary racism as a “recessive tumor” because it has been able to disguise itself behind seemingly “race-neutral” policies (p. 350). Further, Bonilla-Silva (2006) terms contemporary racism “color-blind racism” because many people in society assert that they “don’t see any color, just people” (p. 1). As such, the U.S. social structure, influenced by historical and contemporary racism, has constrained opportunities for Black and Hispanic adults, thereby predisposing them to greater stress exposure.

Weathering, a stress hypothesis, emphasizes that racial and ethnic minorities are more likely to “experience early health deterioration as a consequence of the cumulative impact of repeated experience with social or economic adversity and political marginalization” (Geronimus et al., 2006, p. 826). Further, the weathering hypothesis specifies that racial and ethnic minorities, especially Black adults, acquire health conditions and experience health deterioration at a younger age due to long-term stress exposure (Geronimus et al., 2006).

Based on CIT and the weathering thesis, we formulate three main hypotheses.

H1. *U.S.-born Black, U.S.-born Hispanic, and foreign-born Hispanic adults have higher levels of stress exposure and rates of disability onset than U.S.-born White adults.*

H2. *Stress exposure is associated with a higher risk of disability, assessed in (a) cross-sectional analyses of occurrence (at Wave 1) and (b) longitudinal analyses of incidence over time.*

H3. *Among older adults who did not experience disability by baseline, the influence of CSB on disability onset will occur at earlier ages for minority adults than for U.S.-born White adults.*

## Research Design and Methods

This study uses longitudinal data from the Health and Retirement Study (HRS), a nationally representative, biennial panel study of American adults aged 50 years and older. More information about the design of the HRS is presented by Sonnega and colleagues (2014). Due to the survey design, respondents in the HRS were randomly selected to answer questions for several domains of stress exposure, beginning in the 2006 or 2008 psychosocial questionnaire. To optimize the use of variables from this half-sample questionnaire, we gathered Wave 1 (W1) disability and most covariates in either 2006 or 2008, matching participation in the psychosocial questionnaire, and followed initial respondents prospectively through 2020 (W8).

The analysis sample is limited to respondents who met the following criteria: (a) participated and had nonzero weights (i.e., 50+ years of age, noninstitutionalized) in the 2006/2008 wave including the psychosocial module (*N* = 13,770), (b) self-identified as U.S.-born non-Hispanic White, U.S.-born non-Hispanic Black, U.S.-born Hispanic, or foreign-born Hispanic (*N* = 12,931), (c) scored greater than 6 on cognition at W1 (≤6 indicates presence of dementia on the modified version of the telephone interview for cognitive status (TICS) survey; *N* = 12,498), (d) provided responses to at least 2/3 of the questions within each stress domain (*N* = 11,840), and (e) were not missing on any covariates in the study (*N* = 11,483). In addition, for the longitudinal analysis, respondents had to report no disability prior to or at baseline age to be included (*N* = 8,400). Thus, the analytic sample for studying the occurrence of disability at W1 uses data from 11,483 older adults; and the analytic sample for prospectively studying disability onset over 12 or more years of observation uses data from 8,400 older adults.

### Disability

We measured disability by using RAND’s conceptualization of activity of daily living limitations, which include any difficulty in bathing, eating, dressing, walking across a room, and/or getting in or out of bed (Bugliari et al., 2023). To capture the occurrence of disability, a dummy variable was created using 1994–2006/2008 data (0 = no disability, 1 = disability prior to or at W1).

Our dependent variable for the incidence analysis used information on the respondent’s age, measured in months, to identify age of disability onset. By limiting the sample to those respondents who had no disability prior to or at age reported at W1, we identified those who experienced incident disability (coded 1; 0 = no disability) during the study period (2006/2008–2020). Given that about 73% of respondents never experienced a disability from 2006/2008 to 2020, the variable for age of disability onset was censored. For people who experienced disability, we recorded the age at which they first reported it. For people who did not experience disability, we recorded the last age they were observed in the data. The age of disability onset is thus a duration variable that we used in our survival models in combination with an indicator for censoring (i.e., an indicator for people who did not experience a disability).

### Life-Course Stressors

To assess life-course stressors, we created a composite measure of stress exposure—*cumulative stress burden* (CSB)—which is an average score of all indicators of stress within the domain-specific measures. CSB quantifies levels of stress across many facets of life. All 35 indicators were dichotomized and averaged to create a range from 0 to 1, in which higher levels represent a higher level of CSB.

We also calculated the averages of seven domain-specific stressors. All domain-specific stress measures were standardized to range from 0 to 1, in which values closer to 1 indicate a higher level of stress exposure. Measures of childhood stressors were gathered from the cross-wave Childhood Health and Family Aggregate data set. *Childhood traumatic events* was the average of four experiences prior to the age of 18: (1) parental substance abuse; (2) parental physical abuse; (3) repeated a year of school; and/or (4) got in trouble with the police (Krause et al., 2004). *Childhood financial strain* was composed of three experiences prior to the age of 16: (1) relocated due to financial strain; (2) received financial help from relatives; and/or (3) had a father who was unemployed for several months.

The other domain-specific stress measures were asked of half-samples in the 2006 and 2008 psychosocial questionnaire. *Lifetime traumatic events* were asked by querying if a respondent ever experienced the following: (1) death of a child; (2) natural disaster; (3) fired a weapon in combat; (4) had a partner addicted to drugs or alcohol; (5) was a victim of a physical attack; (6) had a spouse or child with a serious illness or accident; and/or (7) experienced a serious illness or accident oneself (Krause et al., 2004).

*Adult financial strain* was measured by averaging responses of two indicators: (1) satisfaction with the present financial situation (extremely, very, somewhat = 0; not very, not at all = 1) and (2) difficulty meeting monthly payments (not at all, not very, somewhat = 0; very, extremely = 1; Campbell et al., 1976).

*Neighborhood disadvantage* was derived from the following statements: (1) I feel like I don’t belong in this area; (2) vandalism and graffiti are a big problem in this area; (3) most people in this area cannot be trusted; (4) people would be afraid to walk alone in this area after dark; (5) most people in this area are unfriendly; (6) this area is always full of rubbish and litter; (7) if you were in trouble, there is no one in this area who would help you; and/or (8) there are many vacant or deserted houses or storefronts in this area (Cagney et al., 2009). We coded respondents on a 7-point scale in which 0 = strongly disagree and 6 = strongly agree. Respondents who scored a value of “somewhat agree,” “agree,” or “strongly agree” were identified as experiencing the event and given a score of 1 for that specific indicator.

A measure of *everyday discrimination* was developed from asking respondents how often: (1) they are treated with less courtesy or respect; (2) they receive poorer service than others at restaurants and stores; (3) people act as if they think you are not smart; (4) people act as if they are afraid of you; and (5) are you threatened or harassed (Williams et al., 1997). Respondents who never experienced unfair treatment were coded as 0, whereas respondents who reported experiencing one or more instances of discrimination were coded as 1 for that specific indicator.

Finally, the last domain-specific measure captured major experiences of unfair treatment (i.e., *major lifetime discrimination*). Respondents reported whether they had ever been: (1) unfairly dismissed from a job, (2) not hired for a job, (3) denied a promotion, (4) prevented from moving to a neighborhood because a realtor refused to sell/rent to you, (5) denied a bank loan, and/or (6) stopped by the police (Williams et al., 1997). Affirmative responses for each indicator of major lifetime discrimination were coded as 1 and then averaged across all six indicators. For additional details on the stress measures, see Supplementary Table 1.

### Race, Ethnicity, and Nativity

Numerous studies reveal that nativity is associated with many facets of life, including physical functioning (e.g., Hayward et al., 2014). Therefore, we used four binary variables (0,1) to assess race, ethnicity, and nativity: White (reference group), Black, U.S.-born Hispanic, and foreign-born Hispanic adults. We accounted for nativity status among Hispanic respondents only due to the low percentage of White and Black respondents in the HRS born outside of the United States.

### Covariates

We adjusted for characteristics known to influence disability in later life. We used a binary variable to differentiate men and women (0 and 1, respectively). We also adjusted for three adult resources: *marital status* (married vs nonmarried), *education* (years schooling, top coded at 17+), and *household wealth* (tens of thousands of dollars and cube rooted to correct for skewness). Further, we adjusted for five adult health-related indicators. *Depressive symptoms* were measured using an eight-item Center for Epidemiological Studies—Depression (CES-D) score in which a higher score indicates more symptoms (Bugliari et al., 2023). *Self-rated health* ranged from 0 (poor) to 4 (excellent). A self-reported physical activity scale gathered both frequency and intensity of exercise. Participants were asked how often they participated in moderate and vigorous physical activity, with five response categories: never/rarely = 0; 1–3 times per month = 1; once per week = 2; two or more times per week = 3.; and everyday = 4. The scale was created by weighting the type of *physical activity* by intensity (moderate = 1.4, vigorous = 1.8) based on metabolic equivalent recommendations. Possible scores range from 0 (no physical activity) to 12.8 (moderate and vigorous physical activity daily; Latham & Williams, 2015). *Body mass index* (BMI) was based on self-reports and categorized into underweight or normal weight (BMI < 25), overweight (25 ≥ BMI < 30), and obese (BMI ≥ 30). Finally, we adjusted for *functional limitations,* which were gathered by assessing difficulty in the following tasks: walking several blocks; walking one block; climbing several flights of stairs; climbing one flight of stairs; sitting for 2 hr; getting up from a chair; stooping, kneeling, or crouching; and/or pushing or pulling large objects. Although some scholars treat functional limitations and disability as interchangeable, the disablement model specified by Verbrugge and Jette (1994) asserts that these phenomena are distinct. Indeed, a functional limitation is often an early (upstream) precursor of developing physical disability.

### Analytic Strategy

Analyses were conducted in three steps associated with our hypotheses by using Stata/SE 18.0. First, we used *t*-tests and chi square to examine stress exposure across the four groups. Second, logistic regression models were estimated to assess the relationship between stress exposure and occurrence of disability at W1. Third, for the survival analysis we applied Weibull-accelerated failure-time models (WAFT) to examine if CSB and domain-specific stressors were associated with disability onset (i.e., incidence). We used age as the time metric for the longitudinal analysis to interpret the transition to disability as an age-specific incident function (Thiébaut & Bénichou, 2004). The key inputs for the event history analysis were (a) whether a person developed a disability (censoring variable) and (b) the age (measured in months) at which respondents first reported any difficulty with at least one disability. We interpret results from the event history analysis both in terms of the coefficient (β) and time ratio (*e*^β^). Earlier onset of disability is reflected in a negative β (and *e*^β^ < 1) whereas later onset is reflected in a positive β and (*e*^β^ > 1). To test for the moderation of the influence of stressors on disability incidence (*H3*), we added product terms to the models with all covariates. We examined whether CSB and each domain-specific stressor differed by race, ethnicity, and nativity by estimating separate models in which we created product terms for the stressor and the three minority groups compared to White adults. We present interactions for CSB and childhood traumatic events by race, ethnicity, and nativity as these were the two indicators that differed by group. Finally, we conducted several sensitivity analyses that are described in the results section.

## Results

Table 1 displays descriptive statistics by race, ethnicity, and nativity. About 27% of the sample reported a disability by baseline, but there were notable differences across the four groups. Black, U.S.-born Hispanic, and foreign-born Hispanic adults were more likely than White adults to have at least one disability. Of the respondents free of disability at baseline, about 27% developed a disability during the study period (incidence). Black and foreign-born Hispanic adults were more likely than White adults to develop a disability. Moreover, Black, U.S.-born Hispanic, and foreign-born Hispanic adults developed a disability at a younger age than White adults.

**Table 1.** Descriptive Statistics, Total Sample and by Race, Ethnicity, and Nativity

Variables	Range	Total *N* = 11,483	White *n * = 9,280	Black *n* = 1,340	U.S.-born Hispanic *n* = 405	Foreign-born Hispanic *n* = 458
Occurrence of disability	0,1	0.27	0.25	0.36^a^	0.34^b^	0.40^c^
Incident disability^g^	0,1	0.27	0.27	0.30^a^	0.29	0.35^c^
Age of incident disability^g^^,^^h^	52–102	76.97	77.40	75.12^a^	74.36^b^	74.16^c^
Life-course stress						
Cumulative stress burden	0–1	0.17	0.16	0.21^a^	0.19^b^^,^^d^	0.18^c^^,^^e^
Domain-specific stressors						
Childhood traumatic events	0–1	0.10	0.10	0.10	0.14^b^^,^^d^	0.09^f^
Childhood financial strain	0–1	0.18	0.18	0.19	0.24^b^^,^^d^	0.15^c^^,^^e^^,^^f^
Lifetime traumatic events	0–1	0.18	0.18	0.19	0.20^b^	0.17^e^^,^^f^
Adult financial strain	0–1	0.32	0.30	0.44^a^	0.38^b^^,^^d^	0.43^c^^,^^f^
Neighborhood disadvantage	0–1	0.24	0.22	0.34^a^	0.30^b^^,^^d^	0.35^c^^,^^f^
Everyday discrimination	0–1	0.12	0.12	0.15^a^	0.14^b^	0.11^e^^,^^f^
Major lifetime discrimination	0–1	0.08	0.07	0.15^a^	0.09^b^^,^^d^	0.07^e^^,^^f^
Demographics						
Age at baseline	52–100	68.84	69.36	67.06^a^	65.75^b^^,^^d^	66.35^c^
Women	0,1	0.58	0.57	0.65^a^	0.59^d^	0.60^e^
Adult resources						
Married	0,1	0.66	0.69	0.48^a^	0.67^d^	0.64^c^^,^^e^
Education (in years)	0–17	12.81	13.20	12.17^a^	11.03^b^^,^^d^	8.43^c^^,^^e^^,^^f^
Household wealth (cube root in $10,000s)	−6.04–16.09	2.91	3.17	1.76^a^	2.13^b^^,^^d^	1.72^c^^,^^f^
Adult health						
Depressive symptoms	0–8	1.33	1.22	1.58^a^	1.89^b^^,^^d^	2.22^c^^,^^e^^,^^f^
Self-rated health	0–4	2.21	2.30	1.88^a^	1.84^b^	1.60^c^^,^^e^^,^^f^
Physical activity	0–12.8	4.81	4.93	4.16^a^	4.68^d^	4.39^c^
BMI						
Underweight/normal (<25)	0,1	0.29	0.32	0.19^a^	0.19^b^	0.24^c^^,^^e^
Overweight (25 ≥ BMI ≤ 30)	0,1	0.38	0.39	0.33^a^	0.44^b^ ^d^	0.37^f^
Obese (≥30)	0,1	0.32	0.30	0.48^a^	0.38^b^^,^^d^	0.39^c^^,^^e^
Functional limitations	0–8	2.35	2.27	2.72^a^	2.65^b^	2.61^c^

*Notes*: Numbers are means or proportions. The sample size identified in the table is used for most variables and indicates *N* of cases in the occurrence analysis. Significance across subsamples indicated at *p* < .05. BMI = body mass index.

^a^Comparing Black and White adults.

^b^Comparing U.S.-born Hispanic and White adults.

^c^Comparing foreign-born Hispanic and White adults.

^d^Comparing U.S.-born Hispanic and Black adults.

^e^Comparing foreign-born Hispanic and Black adults.

^f^Comparing foreign-born Hispanic and U.S.-born Hispanic adults.

^g^ *N* of cases is different across these rows because the means or proportions are gathered from respondents participating in the incidence analysis (8,400 total respondents; 7,006 White respondents; 852 Black respondents; 266 U.S.-born Hispanic respondents; 276 foreign-born Hispanic respondents).

^h^Age of first disability among those respondents who did not experience a disability by baseline but developed one during the study period.

Among all respondents participating in the occurrence of disability analysis, the average CSB exposure was 0.17 (this equates to about 6 of the 35 total CSB indicators); however, Black, U.S.-born Hispanic, and foreign-born Hispanic adults reported a higher CSB than White adults, with Black adults reporting significantly more stress than the other groups. Examining domain-specific stressors, we also found significant differences across the four groups. For example, Black, U.S.-born Hispanic, and foreign-born Hispanic adults reported higher levels of adult financial strain and neighborhood disadvantage than White adults. U.S.-born Hispanic adults reported the highest level of childhood traumatic events and childhood financial strain. Further, compared to White adults, Black, U.S.-born Hispanic, and foreign-born Hispanic adults had lower levels of socioeconomic status in later life measured by education and household wealth. Black, U.S.-born Hispanic, and foreign-born Hispanic adults had more depressive symptoms, lower levels of self-rated health, higher rates of obesity, and more functional limitations than their White counterparts.

### Disability Occurrence

Table 2 displays the logistic regressions predicting any disability by W1. Model 1 revealed that CSB was associated with higher odds of reporting at least one disability by W1, net of covariates (odds ratio [OR] = 4.93, 95% confidence interval [CI]: 2.95–8.23). Model 2 examined domain-specific stressors and revealed that childhood financial strain, lifetime traumatic events, neighborhood disadvantage, and major lifetime discrimination were associated with higher odds of disability, net of covariates (respectively, OR = 1.22, 95% CI: 1.01–1.46; OR = 1.92, CI: 1.41–2.62; OR = 1.32, CI: 1.01–1.73; OR = 1.64, CI: 1.12–2.41). Black and foreign-born Hispanic adults were more likely than White adults to manifest disability by W1. U.S.-born Hispanic adults did not differ from White adults on W1 disability occurrence.

**Table 2.** Logistic Regression Predicting Disability Occurrence at or Before Age Reported at Wave 1 (*N* = 11,483)

Independent variables	Model 1		Model 2	
	OR	95% CI	OR	95% CI
Demographics				
Black^a^	1.38***	1.17–1.63	1.36***	1.15–1.61
U.S.-born Hispanic^a^	1.25	0.94–1.64	1.24	0.94–1.63
Foreign-born Hispanic^a^	1.62***	1.23–2.11	1.63***	1.25–2.14
Women^b^	0.88*	0.78–0.99	0.88*	0.79–1.00
Age at baseline	1.03***	1.02–1.04	1.03***	1.02–1.04
Life-course stress				
Cumulative stress burden	4.93***	2.95–8.23	—	—
Domain-specific stressors				
Childhood traumatic events	—	—	1.15	0.84–1.58
Childhood financial strain	—	—	1.22*	1.01–1.46
Lifetime traumatic events	—	—	1.92***	1.41–2.62
Adult financial strain	—	—	1.25	0.97–1.63
Neighborhood disadvantage	—	—	1.32*	1.01–1.73
Everyday discrimination	—	—	1.23	0.84–1.81
Major lifetime discrimination	—	—	1.64*	1.12–2.41
Constant	0.02***		0.01***	
Pseudo *R*^2^	0.33		0.34	

*Notes*: OR = odds ratio; CI = confidence interval. Models adjusted for marital status, education, household wealth, depressive symptoms, self-rated health, physical activity, body mass index, and functional limitations.

^a^Reference group is White.

^b^Reference group is men.

*p < .05. **p < .01. ***p < .001.

### CSB and Disability Incidence

**Figure 1.**
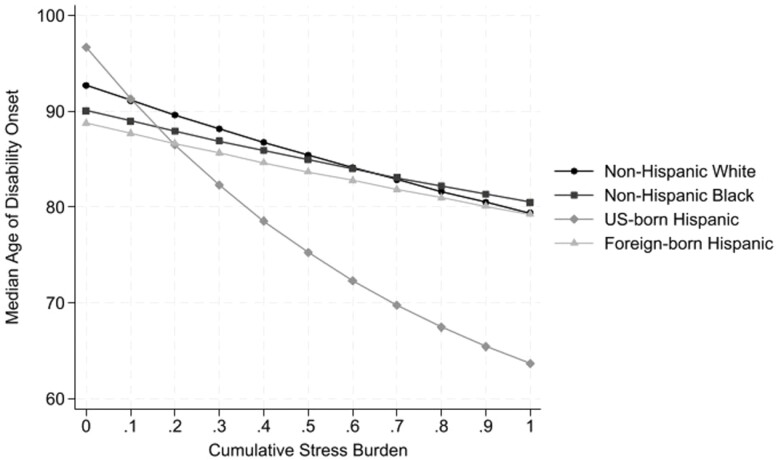
Predicted median age of disability onset by cumulative stress burden and race, ethnicity, and nativity.

To assess the influence of CSB on incident disability, estimates from WAFT models are presented in Table 3. Model 1 included demographics only and revealed that Black, U.S.-born Hispanic, and foreign-born Hispanic adults were more likely to experience the onset of disability at a younger age than White adults (respectively, β = −0.11, β = −0.13, β = −0.15, each *p* < .001). Model 2 included CSB and revealed that persons reporting higher levels of stress were more likely to experience an earlier onset of disability (β = −0.60, *p* < .001). The negative slope revealed that time to disability onset decreased by a factor of 0.55 (exp(−0.60)) for each additional unit increase of CSB. After adjusting for covariates in Model 3, CSB remained significant (β = −0.39*, p* < .001) and there were notable racial and ethnic differences. Black, U.S.-born Hispanic, and foreign-born Hispanic adults experienced a 4% [(1 − *e*^−0.04^) × 100], 7%, and 8% earlier time to onset of disability compared to White adults, respectively. Model 4 examined whether the influence of CSB on the development of disability varied by race, ethnicity, and nativity. We found that CSB was associated with an earlier onset of disability for U.S.-born Hispanic adults compared to White adults (β = −0.86, *p* < .001). As illustrated in Figure 1, U.S.-born Hispanic adults who did not experience any exposure to CSB had the *latest* predicted median age of disability onset. In contrast, higher levels of CSB for U.S.-born Hispanic adults were associated with a much earlier median age of disability onset compared to White adults.

**Table 3.** Weibull-Accelerated Failure-Time Models Associated With Cumulative Stress Burden and the Incidence of Disability (*N* = 8,400)

Independent variables	Model 1			Model 2			Model 3			Model 4		
	β	*SE*	Time ratio (*e*^β^)	β	*SE*	Time ratio (*e*^β^)	β	*SE*	Time ratio (*e*^β^)	β	*SE*	Time ratio (*e*^β^)
Demographics												
Black^a^	−0.11***	0.02	0.90	−0.08***	0.02	0.93	−0.04**	0.02	0.96	−0.06	0.03	0.94
U.S.-born Hispanic^a^	−0.13***	0.03	0.88	−0.12***	0.03	0.89	−0.07*	0.03	0.93	0.09	0.06	1.09
Foreign-born Hispanic^a^	−0.15***	0.03	0.86	−0.14***	0.03	0.87	−0.08**	0.03	0.92	−0.10*	0.05	0.91
Women^b^	−0.01	0.01	0.99	−0.03*	0.01	0.97	−0.01	0.01	0.99	−0.02	0.01	0.99
Cumulative stress burden (CSB)				−0.60***	0.05	0.55	−0.39***	0.05	0.68	−0.37***	0.06	0.69
Covariates												
Married^c^							−0.07***	0.01	0.93	−0.07***	0.01	0.93
Education							−0.01**	0.002	0.99	−0.01**	0.002	0.99
Household wealth							0.03***	0.004	1.03	0.03***	0.004	1.03
Depressive symptoms							−0.02***	0.003	0.98	−0.02***	0.003	0.98
Self-rated health							0.04***	0.01	1.04	0.04***	0.01	1.04
Physical activity							−0.004*	0.002	0.996	−0.004*	0.002	0.996
Overweight^d^							−0.04***	0.01	0.96	−0.04**	0.01	0.96
Obese^d^							−0.13***	0.01	0.88	−0.13***	0.01	0.88
Functional limitations							−0.03***	0.003	0.97	−0.03***	0.003	0.97
Interactions												
CSB × Black										0.10	0.14	1.11
CSB × U.S.-born Hispanic										−0.86***	0.24	0.43
CSB × foreign-born Hispanic										0.09	0.25	1.10
Constant	3.79***			3.89***			3.93***			3.93***		
Likelihood ratio χ^2^	78.17			210.72			754.69			767.92		

*Notes*: *SE* = standard error. A negative β reflects earlier onset of disability (positive, later onset).

^a^Reference group is White.

^b^Reference group is men.

^c^Reference group is not married.

^d^Reference group is underweight/normal BMI.

*p < .05. **p < .01. ***p < .001.

### Domain-Specific Stressors and Disability Incidence

We present parallel models using the domain-specific stressor variables in Table 4. In Model 1, the WAFT models revealed that childhood traumatic events, adult financial strain, everyday discrimination, and major lifetime discrimination were associated with an earlier onset of disability (respectively, β = −0.18, β = −0.27, β = −0.21, β = −0.13, each *p* < .001). Although slightly attenuated, these coefficients remained significant in Model 2 after adjusting for covariates.

**Table 4.** Weibull-Accelerated Failure-Time Models Associated With Domain-Specific Stress and the Incidence of Disability (*N* = 8,400)

Independent variables	Model 1			Model 2			Model 3		
	β	*SE*	Time ratio (*e*^β^)	β	*SE*	Time ratio (*e*^β^)	β	*SE*	Time ratio (*e*^β^)
Demographics									
Black^a^	−0.04*	0.02	0.96	−0.03	0.02	0.97	−0.03	0.02	0.97
U.S.-born Hispanic^a^	−0.10***	0.03	0.90	−0.07**	0.03	0.93	−0.02	0.03	0.98
Foreign-born Hispanic^a^	−0.12***	0.03	0.89	−0.08**	0.03	0.92	−0.09**	0.03	0.91
Women^b^	−0.02	0.01	0.98	−0.01	0.01	0.99	−0.01	0.01	0.99
Domain-specific stressors									
Childhood traumatic events	−0.18***	0.03	0.83	−0.16***	0.03	0.86	−0.15***	0.03	0.86
Childhood financial strain	−0.02	0.02	0.98	−0.01	0.02	0.99	−0.01	0.02	0.99
Lifetime traumatic events	−0.01	0.03	0.99	0.01	0.03	1.01	0.003	0.03	1.003
Adult financial strain	−0.27***	0.02	0.77	−0.17***	0.02	0.84	−0.17***	0.02	0.84
Neighborhood disadvantage	−0.03	0.03	0.97	−0.01	0.03	0.99	−0.01	0.03	0.99
Everyday discrimination	−0.21***	0.04	0.81	−0.15***	0.04	0.86	−0.15***	0.04	0.86
Major lifetime discrimination	−0.13***	0.04	0.88	−0.13***	0.04	0.87	−0.14***	0.04	0.87
Covariates									
Married^c^				−0.06***	0.01	0.94	−0.06***	0.01	0.94
Education				−0.01**	0.002	0.99	−0.01**	0.002	0.994
Household wealth				0.02***	0.004	1.02	0.02***	0.004	1.02
Depressive symptoms				−0.02***	0.003	0.98	−0.02***	0.003	0.98
Self-rated health				0.04***	0.01	1.04	0.04***	0.01	1.04
Physical activity				−0.003	0.002	0.997	−0.003	0.002	0.997
Overweight^d^				−0.03**	0.01	0.97	−0.04**	0.01	0.97
Obese^d^				−0.12***	0.01	0.89	−0.12***	0.01	0.89
Functional limitations				−0.03***	0.003	0.97	−0.03***	0.003	0.97
Interactions									
Childhood traumatic events × Black							0.01	0.09	1.01
Childhood traumatic events × U.S.-born Hispanic							-0.40**	0.13	0.67
Childhood traumatic events × foreign-born Hispanic							0.10	0.14	1.11
Constant	3.92***			3.96***			3.96***		
Likelihood ratio χ^2^	383.68			840.34			849.09		

*Notes*: *SE* = standard error. A negative β reflects earlier onset of disability (positive, later onset).

^a^Reference group is White.

^b^Reference group is men.

^c^Reference group is not married.

^d^Reference group is underweight/normal BMI.

*p < .05. **p < .01. ***p < .001.

Model 3 displays the interaction between childhood traumatic events and each of the three groups, but only the product term with U.S.-born Hispanic adults had a distinct effect on disability onset. This model revealed that childhood traumatic events had a more detrimental influence on later-life disability for U.S.-born Hispanic adults than White adults (β = −0.40, *p* < .01), reflected in a 33% [(1 − *e*^–0.40^) × 100] earlier time to onset.

### Sensitivity Analyses

We conducted sensitivity analyses to examine the robustness of our results. First, due to an erroneous skip pattern in the 2018 HRS survey affecting disability questions, responses to several of the questions were imputed. We completed identical longitudinal analyses using 2016 as our final wave of data collection and had parallel conclusions to those based on the imputed data. Second, this study examined the first manifestation of disability; however, we also estimated zero-inflated negative binomial models investigating the count of disability at W1, 6 years later, and 12 years later. Similar to results from the logistic regression and WAFT models presented, we found that CSB was associated with a higher probability of disability and that the main conclusions were unchanged by estimation with the count data models. Third, we stratified the WAFT models by race, ethnicity, and nativity and present findings in Supplementary Tables 2 and 3.

## Discussion and Implications

Distinct from prior studies of stress and disability, the present study examined CSB as well as the effects of domain-specific stressors in a diverse national sample to understand the significance of different types of stress on later-life disability. Whether examining cumulative or domain-specific stressors, there were substantial disparities in stress exposure. Moreover, life-course stressors elevated the risk of disability in later life, especially for U.S.-born Hispanic adults.

### Stress Exposure and Disability

Addressing our first hypothesis, we found significant differences in stress exposure and disability across the life course by race, ethnicity, and nativity. Results from our descriptive analysis revealed that Black, U.S.-born Hispanic, and foreign-born Hispanic adults experienced greater CSB than White adults. Black adults experienced the most stressors across the life course, a finding consistent with prior studies (Boen & Hummer, 2019; Sternthal et al., 2011). Racial, ethnic, and nativity differences in domain-specific stress exposure were more nuanced, including that (1) U.S.-born Hispanic adults reported the highest levels of childhood traumatic events and childhood financial strain, (2) White adults experienced the lowest levels of adult financial strain and neighborhood disadvantage, and (3) Black adults reported significantly more experiences of lifetime discrimination than White or Hispanic adults. These findings provide support for Hypothesis 1 and theories related to weathering and cumulative inequality such that people with racial or ethnic minority status have greater exposure to negative experiences across the life course (Ferraro et al., 2009; Geronimus et al., 2006).

Consistent with previous literature, Black, U.S.-born Hispanic, and foreign-born Hispanic adults were at higher risk of experiencing disability than White adults (Hayward et al., 2014; Vásquez et al., 2020). Among respondents free of disability at W1 that developed at least one disability during the study period, Black, U.S.-born Hispanic, and foreign-born Hispanic adults experienced the onset of disability at an earlier age. These results support the notion of “weathering” such that minority adults experienced health deterioration, measured by disability onset, at a younger age (Geronimus et al., 2006), but are inconsistent with the Hispanic paradox because foreign-born Hispanic adults were at higher risk than White adults of developing at least one disability at an earlier age.

### Disentangling the Relationship Between Stress Exposure and Disability

Investigating our second hypothesis revealed the long-term health effects of stress exposure on physical functioning. CSB and major lifetime discrimination were associated with disability in the cross-sectional *and* the longitudinal analyses. We speculate that major lifetime discrimination identifies existential instances of discrimination that may be more disruptive to *core* areas of one’s life including employment and residential contexts. Williams and Mohammed (2009) argue that institutional discrimination in these contexts can “trigger elevated exposure to additional stressors, especially those linked to social and economic deprivation” (p. 15). Thus, the potency of major discrimination on later-life health and disability merits study.

Our domain-specific cross-sectional and longitudinal analyses revealed additional unique effects between different types of stress exposure and disability in later life that warrant further exploration. Childhood financial strain, neighborhood disadvantage, and lifetime traumatic events were associated with a higher risk of disability at W1 but were not associated with an earlier onset of disability throughout the study period. According to CIT, disadvantages increase exposure to risk (Ferraro & Shippee, 2009). We suggest that exposure to financial difficulties during childhood may be particularly detrimental because it may limit one’s access to early-life opportunities and resources and, concomitantly, hinder their physical capabilities earlier.

In contrast, childhood traumatic events, adult financial strain, and everyday discrimination were not associated with a higher likelihood of having a disability at W1; however, respondents who experienced higher levels of these specific stressors experienced the onset of disability at an earlier age during the study period. These findings underscore the importance of “weathering” as a stress hypothesis. More specifically, results for childhood trauma provide evidence of a latency effect, such that these early-life conditions may “get under the skin,” and the influence may not manifest until later life, akin to a time-release capsule. Moreover, even though everyday discrimination may not involve a life-altering experience, it is demeaning to be treated with less courtesy and respect and to receive poorer service in everyday encounters. We speculate that over the long haul, the degrading experiences of structured inequalities that are evident in everyday discrimination are insidious for physical functioning. Taken together, these results show that a lifetime filled with stress exposure takes a toll on physical functioning, providing ample evidence for Hypothesis 2.

### Disparities in the Relationship Between Stress Exposure and Disability

Our third hypothesis involved examining the influence of both CSBand domain-specific stressors on disability while also testing for the moderating effect of race, ethnicity, and nativity. Compared to White adults, U.S.-born Hispanic adults who experienced greater CSB—especially childhood trauma—faced an earlier onset of disability. In other words, accumulated exposure to negative events across the life course and trauma during childhood was especially detrimental among U.S.-born Hispanic adults. Moderation was not observed for Black and foreign-born Hispanic adults, but moderation for U.S.-born Hispanics provides partial support for Hypothesis 3.

We offer two potential explanations for why U.S.-born Hispanic adults were especially hard hit by the negative effects of childhood traumatic experiences. First, compared to White, Black, and foreign-born Hispanic adults, U.S.-born Hispanic adults had the highest levels of childhood traumatic events. Although high levels of stress exposure do not necessarily lead to worse health outcomes, examining the specific indicators of childhood trauma showed that a higher percentage of U.S.-born Hispanic adults were more likely than any other group to report physical abuse by a parent, and they also were more likely than White and foreign-born Hispanic adults to report trouble with the police during childhood. The gravity of these existential stressors may be one reason for their long-term impact (Slopen et al., 2016). This finding underscores the need to focus on coping and support services among U.S.-born Hispanic adults who had traumatic childhood experiences, perhaps because they were called on at a young age to help their parents navigate U.S. social service institutions. For instance, some U.S.-born Hispanic children accompany their parents to social services agencies and government entities to provide translation for their parents with limited English fluency. As such, those young people get an open view of family disadvantage and the difficulty of accessing social resources and services.

Second, U.S.-born Hispanic adults may face a lifetime of ethnic marginalization that leads to disability. Compared to foreign-born Hispanic adults, “duration of residence correlates not with increased integration, but with exposure to the detrimental effects of social and economic disadvantage” (Angel & Whitfield, 2007, p. 5). U.S.-born Hispanic adults may experience these barriers and some degree of marginalization from the moment they are born. Consistent with Brown (2018), we found that early health deterioration is more pronounced in minority populations, especially U.S.-born Hispanic Americans.

Although we found that CSB, adult financial strain, everyday discrimination, and major lifetime discrimination were associated with earlier onset of disability, we were surprised that we did not find evidence for moderation by race, ethnicity, and nativity  within these domains. We offer two plausible reasons for why there was no evidence of moderation among Black and foreign-born Hispanic adults. First, although Black adults experienced higher levels of stress than White persons for most domains, some research reveals that Black and Hispanic adults appraise chronic stressors as less upsetting (Brown et al., 2020). Thus, the distinction between stress exposure and stress appraisal may be informative for future studies of health disparities. In analyses stratified by race, ethnicity, and nativity, we learned that CSB was not associated with an earlier onset of disability among Black and foreign-born Hispanic respondents. We speculate that these findings may be due to differing levels of appraisal by Black people facing a lifetime of adversity (Brown et al., 2020). Perhaps less reactivity to stressful experiences is beneficial for later-life physical functioning. It is also possible that foreign-born Hispanic respondents were health advantaged at the time of immigration and were able to retain their physical function.

Second, at least one study showed that discrimination based on changing characteristics such as age and weight was more consequential to health than discrimination based on more stable characteristics such as race and sex (Sutin et al., 2015). Although we focused on moderation by race, ethnicity, and nativity, perhaps the effect of the stressors varies by other status characteristics, especially age.

### Limitations

Although this study makes important contributions to the growing literature on disparities in stress and physical health, there are two notable limitations. First, we examined multiple domains of stress, but there are other potential stressors that are influential to health disparities such as relationship quality and unequal access to and treatment within healthcare services. Also, foreign-born Hispanic adults may be particularly vulnerable to acculturation stressors during adulthood, including language barriers and living outside one’s home country, which were not assessed in this study (Boen & Hummer, 2019). Second, this study used valuable life course data, but the analysis is limited by the crude delineation of time reflected in the measures. It is useful to have data separated into the periods of childhood and adulthood, but a more precise delineation of timing within each period may reveal sequences of experiences that would advance the literature in notable ways.

## Conclusions

This study illustrates the importance of a life course perspective for understanding disparities in later-life disability among White, Black, U.S.-born Hispanic, and foreign-born Hispanic older adults. There are major differences in stress exposure across those four groups, and those differences have long-lasting effects on disability in later life. Although many stressors had an independent influence on disability, we also found evidence for the moderation of that relationship. We discovered that U.S.-born Hispanic adults who experienced childhood traumatic events faced an earlier transition to disability during later life than their White counterparts. Although one may reason that the effect of childhood stressors is likely to be exhausted or resolved during early and middle age, our findings revealed that the influence of early trauma on physical function among U.S.-born Hispanic persons lasts for decades. As such, it is important to also acknowledge that nativity influences stratification processes associated with disparities in racial and ethnic health trajectories. Given recent immigration trends, reducing these disparities in disability during later life will require culturally sensitive interventions to ameliorate the long-lasting historical and structural disadvantages that these groups have faced.

## Supplementary Material

igae039_suppl_Supplementary_Tables

## Data Availability

The data underlying this article are available from the University of Michigan Health and Retirement Study. The data sets were derived from sources in the public domain: Health and Retirement Study (https://hrs.isr.umich.edu/documentation/data-descriptions). The study was not preregistered.
