# Supplementary material for: Disparities in Stress Exposure and Later-Life Disability
Source: Innov Aging. 2024 Apr 25;8(5):igae039. doi: 10.1093/geroni/igae039 (PMC11134293; doi:10.1093/geroni/igae039)
Supplement: igae039_suppl_Supplementary_Tables [file igae039_suppl_supplementary_tables.docx]

***Innovation in Aging* Supplementary Material: Madison R. Sauerteig-Rolston, & Kenneth F. Ferraro. Disparities in Stress Exposure and Later-Life Disability.**

**Supplemental Table 1**. Domain-specific stressor and cumulative stress burden measure construction

| Stressor Measure | Question | Item coding | Scale coding^a^ | Binary coding^b^ |
| --- | --- | --- | --- | --- |
| **Childhood Trauma** | Before you were 18… |  | (Sum of items)/4 |  |
|  | Did you have to do a year of school over again? | 0=no, 1=yes |  | 0=no, 1=yes |
|  | Were you ever in trouble with the police? | 0=no, 1=yes |  | 0=no, 1=yes |
|  | Did either of your parents’ drink or use drugs so often that it caused problems in the family? | 0=no, 1=yes |  | 0=no, 1=yes |
|  | Were you ever physically abused by either of your parents? | 0=no, 1=yes |  | 0=no, 1=yes |
| **Childhood Financial Hardship** | Before you were 16… |  | (Sum of items)/3 |  |
|  | Did financial difficulties ever cause you or your family to move to a different place? | 0=no, 1=yes |  | 0=no, 1=yes |
|  | Was there a time when you or your family received financial help from relatives because of financial difficulties? | 0=no, 1=yes |  | 0=no, 1=yes |
|  | Was there a time of several months or more when your father had no job? | 0=no, 1=yes |  | 0=no, 1=yes |
| **Lifetime trauma** | Has a child of yours ever died? | 0=no, 1=yes | (Sum of items)/7 | 0=no, 1=yes |
|  | Have you ever been in a major fire, flood, earthquake, or other natural disaster? | 0=no, 1=yes |  | 0=no, 1=yes |
|  | Have you ever fired a weapon in combat or been fired upon in combat? | 0=no, 1=yes |  | 0=no, 1=yes |
|  | Has your spouse/partner/child ever been addicted to drugs or alcohol? | 0=no, 1=yes |  | 0=no, 1=yes |
|  | Were you the victim of a physical attack or assault? | 0=no, 1=yes |  | 0=no, 1=yes |
|  | Did you ever have a life-threatening illness or accident? | 0=no, 1=yes |  | 0=no, 1=yes |
|  | Did your spouse or a child of yours ever have a life-threatening illness or accident? | 0=no, 1=yes |  | 0=no, 1=yes |
| **Adult financial strain** | How satisfied are you with your present financial situation? | 0=extremely; 1= very; 2= somewhat; 3= not very; 4= not at all | (Average of items)/2 | 0=extremely, very, somewhat  1= not very, not at all |
|  | How difficult is it for you to meet monthly payments? | 0= not at all; 1= not very; 2= somewhat; 3= very; 4= extremely |  | 0=not at all, not very, somewhat  1= very, extremely |
| **Neighborhood Disadvantage** | I feel like I don’t belong in this area. | 0=strongly disagree; 1=disagree; 2= somewhat disagree; 3=neither agree nor disagree; 4= somewhat agree; 5= agree; 6= strongly agree | (Average of items)/6 | 0=strongly disagree, disagree, somewhat disagree, neither agree nor disagree; 1=somewhat agree, agree, strongly agree |
|  | Vandalism and graffiti are a big problem in this area. | 0=strongly disagree; 1=disagree; 2= somewhat disagree; 3=neither agree nor disagree; 4= somewhat agree; 5= agree; 6= strongly agree |  | 0=strongly disagree, disagree, somewhat disagree, neither agree nor disagree; 1=somewhat agree, agree, strongly agree |
|  | Most people in this area can’t be trusted. | 0=strongly disagree; 1=disagree; 2= somewhat disagree; 3=neither agree nor disagree; 4= somewhat agree; 5= agree; 6= strongly agree |  | 0=strongly disagree, disagree, somewhat disagree, neither agree nor disagree; 1=somewhat agree, agree, strongly agree |
|  | People would be afraid to walk alone in this area after dark. | 0=strongly disagree; 1=disagree; 2= somewhat disagree; 3=neither agree nor disagree; 4= somewhat agree; 5= agree; 6= strongly agree |  | 0=strongly disagree, disagree, somewhat disagree, neither agree nor disagree; 1=somewhat agree, agree, strongly agree |
|  | Most people in this area are unfriendly. | 0=strongly disagree; 1=disagree; 2= somewhat disagree; 3=neither agree nor disagree; 4= somewhat agree; 5= agree; 6= strongly agree |  | 0=strongly disagree, disagree, somewhat disagree, neither agree nor disagree; 1=somewhat agree, agree, strongly agree |
|  | This area is always full of rubbish and litter. | 0=strongly disagree; 1=disagree; 2= somewhat disagree; 3=neither agree nor disagree; 4= somewhat agree; 5= agree; 6= strongly agree |  | 0=strongly disagree, disagree, somewhat disagree, neither agree nor disagree; 1=somewhat agree, agree, strongly agree |
|  | If you were in trouble, there is no one in this area who would help you. | 0=strongly disagree; 1=disagree; 2= somewhat disagree; 3=neither agree nor disagree; 4= somewhat agree; 5= agree; 6= strongly agree |  | 0=strongly disagree, disagree, somewhat disagree, neither agree nor disagree; 1=somewhat agree, agree, strongly agree |
|  | There are many vacant or deserted houses or storefronts in this area. | 0=strongly disagree; 1=disagree; 2= somewhat disagree; 3=neither agree nor disagree; 4= somewhat agree; 5= agree; 6= strongly agree |  | 0=strongly disagree, disagree, somewhat disagree, neither agree nor disagree; 1=somewhat agree, agree, strongly agree |
| **Everyday Discrimination** | How often…. |  |  |  |
|  | Are you treated with less courtesy or respect? | 0=never; 1=less than once a year; 2= a few times a year; 3= a few times a month; 4= at least once a week; 5= almost everyday | (Average of items)/5 | 0=never;  1= at least once |
|  | Do you receive poorer service than others at restaurants or stores? | 0=never; 1=less than once a year; 2= a few times a year; 3= a few times a month; 4= at least once a week; 5= almost everyday |  | 0=never;  1= at least once |
|  | Do people act as if they think you are not smart? | 0=never; 1=less than once a year; 2= a few times a year; 3= a few times a month; 4= at least once a week; 5= almost everyday |  | 0=never;  1= at least once |
|  | Do people act as if they are afraid of you? | 0=never; 1=less than once a year; 2= a few times a year; 3= a few times a month; 4= at least once a week; 5= almost everyday |  | 0=never;  1= at least once |
|  | Are you threatened or harassed? | 0=never; 1=less than once a year; 2= a few times a year; 3= a few times a month; 4= at least once a week; 5= almost everyday |  | 0=never;  1= at least once |
| **Major Lifetime Discrimination** | Have you ever been… |  | (Sum of items)/6 |  |
|  | Unfairly dismissed from a job? | 0=no, 1=yes |  | 0=no, 1=yes |
|  | Not hired for a job? | 0=no, 1=yes |  | 0=no, 1=yes |
|  | Denied a promotion? | 0=no, 1=yes |  | 0=no, 1=yes |
|  | Prevented from moving to a neighborhood because a realtor refused to sell/rent to you? | 0=no, 1=yes |  | 0=no, 1=yes |
|  | Denied a bank loan? | 0=no, 1=yes |  | 0=no, 1=yes |
|  | Stopped by police? | 0=no, 1=yes |  | 0=no, 1=yes |
| ^a^Scale coding of indicators within the domain-specific stressors. Each domain-specific stressor is standardized to be on a scale of 0 to 1.  ^b^Binary coding of each individual stressor in the cumulative stress measure. | | | | |

| **Supplemental Table 2.** Weibull accelerated failure-time models associated with cumulative stress burden and the incidence of disability, stratified by race, ethnicity, and nativity (N=8,400) | | | | | | | | | | | | |
| --- | --- | --- | --- | --- | --- | --- | --- | --- | --- | --- | --- | --- |
| Independent Variables | White  (n=7,006) | | | Black  (n= 852) | | | US-born Hispanic (n=266) | | | Foreign-born Hispanic  (n=276) | | |
|  | β | SE | Time ratio (*e*^β^) | β | SE | Time ratio (*e*^β^) | β | SE | Time ratio (*e*^β^) | β | SE | Time ratio (*e*^β^) |
| *Demographics* |  |  |  |  |  |  |  |  |  |  |  |  |
| Women^a^ | -0.01 | 0.01 | 0.99 | -0.09* | 0.04 | 0.92 | 0.06 | 0.07 | 1.06 | -0.05 | 0.08 | 0.95 |
| CSB | -0.37*** | 0.05 | 0.69 | -0.25 | 0.16 | 0.78 | -1.26*** | 0.30 | 0.29 | -0.62 | 0.33 | 0.54 |
| *Covariates* |  |  |  |  |  |  |  |  |  |  |  |  |
| Married^b^ | -0.07*** | 0.01 | 0.94 | -0.09* | 0.04 | 0.92 | -0.08 | 0.08 | 0.93 | -0.11 | 0.08 | 0.89 |
| Education | -0.01*** | 0.002 | 0.99 | -0.01 | 0.01 | 0.99 | -0.01 | 0.01 | 0.99 | 0.02* | 0.01 | 1.02 |
| Household wealth | 0.03*** | 0.004 | 1.03 | 0.02 | 0.01 | 1.02 | 0.03 | 0.02 | 1.03 | 0.03 | 0.02 | 1.03 |
| Depressive symptoms | -0.02*** | 0.003 | 0.98 | -0.03** | 0.01 | 0.97 | -0.02 | 0.02 | 0.98 | -0.04** | 0.02 | 0.96 |
| Self-rated health | 0.03*** | 0.01 | 1.04 | 0.06** | 0.02 | 1.07 | 0.08 | 0.04 | 1.08 | 0.01 | 0.04 | 1.01 |
| Physical activity | -0.002 | 0.002 | 0.998 | -0.01 | 0.01 | 0.99 | -0.002 | 0.01 | 0.998 | -0.01 | 0.01 | 0.99 |
| Overweight^c^ | -0.05*** | 0.01 | 0.96 | 0.07 | 0.04 | 1.08 | -0.15 | 0.10 | 0.86 | -0.21* | 0.09 | 0.81 |
| Obese^c^ | -0.13*** | 0.01 | 0.88 | -0.08 | 0.04 | 0.92 | -0.21* | 0.10 | 0.81 | -0.35*** | 0.09 | 0.71 |
| Functional limitations | -0.03*** | 0.003 | 0.97 | -0.03** | 0.01 | 0.97 | -0.02 | 0.02 | 0.98 | -0.001 | 0.02 | 0.999 |
| Constant | 3.96*** |  |  | 3.89*** |  |  | 4.03*** |  |  | 3.95*** |  |  |
| *Likelihood Ratio χ^2^* | 568.06 |  |  | 85.88 |  |  | 47.06 |  |  | 38.46 |  | |
| Notes: A negative β reflects earlier onset of disability (positive, later onset). SE = standard error.  ^a^Reference group is men. ^b^Reference group is not married. ^c^Reference group is underweight/normal BMI.  **p* < .05; ***p* < 0.01; ****p* < .001. | | | | | | | | | | | | |

| **Supplemental Table 3.** Weibull accelerated failure-time models associated with domain-specific stress and the incidence of disability, stratified by race, ethnicity, and nativity (N=8,400) | | | | | | | | | | | | |
| --- | --- | --- | --- | --- | --- | --- | --- | --- | --- | --- | --- | --- |
| Independent Variables | White  (n=7,006) | | | Black  (n= 852) | | | US-born Hispanic (n=266) | | | Foreign-born Hispanic  (n=276) | | |
|  | β | SE | Time ratio (*e*^β^) | β | SE | Time ratio (*e*^β^) | β | SE | Time ratio  (*e*^β^) | β | SE | Time ratio (*e*^β^) |
| *Demographics* |  |  |  |  |  |  |  |  |  |  |  |  |
| Women^a^ | -0.01 | 0.01 | 0.99 | -0.09* | 0.04 | 0.91 | 0.03 | 0.07 | 1.03 | -0.05 | 0.08 | 0.95 |
| *Domain-specific stressors* |  |  |  |  |  |  |  |  |  |  |  |  |
| Childhood traumatic events | -0.16*** | 0.03 | 0.85 | -0.23* | 0.10 | 0.80 | -0.52** | 0.16 | 0.60 | -0.04 | 0.22 | 0.96 |
| Childhood financial strain | 0.01 | 0.02 | 1.01 | -0.07 | 0.05 | 0.94 | -0.15 | 0.09 | 0.86 | -0.12 | 0.14 | 0.89 |
| Lifetime traumatic events | 0.02 | 0.03 | 1.02 | 0.07 | 0.10 | 1.07 | -0.25 | 0.17 | 0.78 | -0.33 | 0.20 | 0.72 |
| Adult financial strain | -0.17*** | 0.03 | 0.85 | -0.11 | 0.08 | 0.89 | -0.16 | 0.16 | 0.86 | -0.33* | 0.14 | 0.72 |
| Neighborhood disadvantage | -0.01 | 0.03 | 0.99 | -0.02 | 0.07 | 0.99 | 0.04 | 0.16 | 1.04 | 0.07 | 0.15 | 1.07 |
| Everyday discrimination | -0.16*** | 0.04 | 0.85 | 0.03 | 0.11 | 1.03 | -0.51** | 0.18 | 0.60 | -0.50 | 0.26 | 0.60 |
| Major lifetime discrimination | -0.16*** | 0.04 | 0.85 | -0.12 | 0.09 | 0.89 | -0.01 | 0.22 | 0.99 | 0.25 | 0.31 | 1.28 |
| *Covariates* |  |  |  |  |  |  |  |  |  |  |  |  |
| Married^b^ | -0.06*** | 0.01 | 0.95 | -0.08* | 0.04 | 0.93 | -0.06 | 0.08 | 0.94 | -0.10 | 0.08 | 0.91 |
| Education | -0.01*** | 0.002 | 0.99 | -0.01 | 0.01 | 0.995 | -0.02 | 0.01 | 0.98 | 0.02* | 0.01 | 1.02 |
| Household wealth | 0.02*** | 0.004 | 1.02 | 0.01 | 0.01 | 1.01 | 0.03 | 0.02 | 1.03 | 0.02 | 0.03 | 1.03 |
| Depressive symptoms | -0.01*** | 0.003 | 0.99 | -0.03** | 0.01 | 0.97 | -0.01 | 0.02 | 0.99 | -0.04** | 0.02 | 0.96 |
| Self-rated health | 0.03*** | 0.01 | 1.03 | 0.07** | 0.02 | 1.07 | 0.08* | 0.04 | 1.08 | 0.001 | 0.04 | 1.00 |
| Physical activity | -0.001 | 0.002 | 0.999 | -0.01* | 0.01 | 0.99 | -0.002 | 0.01 | 0.998 | -0.01 | 0.01 | 0.99 |
| Overweight^c^ | -0.04** | 0.01 | 0.96 | 0.08 | 0.04 | 1.09 | -0.19 | 0.10 | 0.83 | -0.23* | 0.09 | 0.80 |
| Obese^c^ | -0.12*** | 0.01 | 0.89 | -0.07 | 0.04 | 0.93 | -0.24* | 0.11 | 0.79 | -0.39*** | 0.10 | 0.68 |
| Functional limitations | -0.03*** | 0.003 | 0.97 | -0.03** | 0.01 | 0.97 | -0.03 | 0.02 | 0.97 | 0.01 | 0.02 | 1.01 |
| Constant | 4.00*** |  |  | 3.90*** |  |  | 4.14*** |  |  | 4.09*** |  |  |
| *Likelihood Ratio χ^2^* | 652.65 |  |  | 95.35 |  |  | 57.79 |  |  | 49.10 |  | |

Notes: A negative β reflects earlier onset of disability (positive, later onset). SE = standard error.

^a^Reference group is men. ^b^Reference group is not married. ^c^Reference group is underweight/normal BMI.

**p* < .05; ***p* < 0.01; ****p* < .001.
